# Ferrocene‐Based Sensors for Green Analytical Applications in Environmental, Biomedical, Industrial, and Food Monitoring

**DOI:** 10.1002/open.202500521

**Published:** 2026-04-21

**Authors:** Ala'a Al‐Akhras, Asma Ghazzy, Zainab Zakaraya, Ala'a S. Shraim, Obada A. Sibai, Laila Alsabbagh, Deeb Taher

**Affiliations:** ^1^ Chemistry Department Faculty of Science Jerash University Jerash Jordan; ^2^ Faculty of Pharmacy Al‐Ahliyya Amman University Amman Jordan; ^3^ Department of Medical Laboratory Sciences Allied Medical Sciences Al‐Ahliyya Amman University Amman Jordan; ^4^ Department of Pharmacy, Faculty of Pharmacy Al‐Zaytoonah University of Jordan Amman Jordan; ^5^ Department of Orthopedic Surgery King Abdullah Bin Abdulaziz University Hospital Riyadh Saudi Arabia; ^6^ Department of Chemistry, School of Science University of Jordan Amman Jordan

**Keywords:** biomarker detection, eco‐friendly sensors, ferrocene, pollution monitoring, sustainable technology

## Abstract

Ferrocene derivatives represent a transformative class of molecular sensors, combining exceptional electrochemical stability with tunable redox characteristics that enable precise detection across diverse analytical challenges in environmental, medical, and industrial applications. This comprehensive review examines the expanding applications of ferrocene‐based sensors (FBSs) across critical analytical domains. In environmental monitoring, these sensors achieve remarkable specificity for heavy metals and pesticides, with detection limits reaching nanomolar concentrations, thereby advancing pollution control strategies and ecosystem protection initiatives. In the medical field, FBSs detect biomarkers in biological fluids for early disease diagnosis, including the identification of enzymes and proteins crucial for understanding biological processes. They play a vital role in industrial monitoring by detecting volatile organic compounds (VOCs) to maintain workplace safety and environmental compliance. Furthermore, FBSs are applied to food safety by detecting contaminants, thereby safeguarding public health. These applications highlight the potential of FBSs as innovative tools for advancing sustainability, green chemistry, and eco‐friendly technologies.

## 1 Introduction

The rapid advancement of molecular sensor (MS) technology has catalyzed significant improvements in electrochemical detection systems, creating an urgent demand for redox‐active receptors that combine synthetic accessibility with cost‐effectiveness. Ferrocene derivatives have emerged as exceptionally promising candidates, offering a unique combination of robust electrochemical properties and remarkable functional group versatility that enables tailored sensor design for specific analytical targets. Extensive research has established ferrocene compounds as highly effective molecular sensors, with their exceptional corrosion resistance and thermal stability enabling reliable operation in challenging environmental conditions. These compounds exhibit remarkable analytical versatility, demonstrating sensitive responses to diverse analytes, including gaseous species, pH variations, and temperature fluctuations, making them ideal platforms for multiparameter sensing applications [1, 2, 3, 4, 5, 6, 7, 8, 9, 10, 11, 12].

Historically, ferrocene synthesis has relied on water‐sensitive reagents and volatile organic solvents (such as diethyl ether or tetrahydrofuran), generating substantial amounts of inorganic byproducts and producing large quantities of solvent waste, raising both environmental and safety concerns. In contrast, there are many newer, greener routes being developed that greatly reduce the environmental impact of ferrocene synthesis. The two most notable new green routes include solvent‐free and mechanochemistry routes to ferrocene production; these routes require very little solvent and less energy than traditional synthesis routes to produce high yields [13, 14], as well as ultrasonic‐assisted methods to prepare ferrocene derivatives, where they can also decrease the time it takes to react and the amount of energy required during the synthesis process to make ferrocene derivatives compared to the traditional thermal processes [15]. All of this research indicates that the current trend is away from the use of the traditional solvent‐intensive route of ferrocene synthesis and toward the use of more environmentally friendly synthetic routes. This trend will be important in the evaluation of ferrocene‐based systems as possible sustainable technologies for sensor applications.

Ferrocene compounds have been applied as sensors in a variety of applications, such as environmental monitoring, food safety, and biomedical applications (Table 1). For example, ferrocene compounds have been used as sensors to monitor trace amounts of gases, such as H_2_ and CO_2_, in the environment. They have also been used as pH sensors for food safety purposes, allowing for the rapid detection of pH changes in food products. In the field of biomedical diagnostics, FBSs have been used for the detection of various analytes, such as glucose and cholesterol [22, 23, 24, 25, 26, 27, 28, 29, 30, 31, 32].

**TABLE 1:** Influence of a ferrocene‐based sensor on their applications.

Influence on Sensing Properties	Example of Ferrocene‐Based Sensor	Application	Ref
Stability improves robustness and durability	Ferrocene‐modified screen‐printed electrode for the detection of lead ions	Environmental monitoring	[16]
Redox activity improves sensitivity	Ferrocene‐modified electrode for the detection of hydrogen peroxide	Biosensing	[17, 18]
Conductivity improves sensitivity	Ferrocene‐doped graphene for the detection of dopamine	Medical diagnostics	[19]
Catalytic activity improves selectivity	Ferrocene‐modified titanium dioxide for the detection of ethanol	Food safety	[20, 21]

The development of ferrocene‐based sensors is an increasingly popular field of study due to the versatility of these materials as analytical systems that can undergo reversible oxidation/reduction reactions, show high chemical stability, and possess tunable electrical properties, allowing for the most efficient possible transduction of signals in both electrochemical and optical sensing devices [25, 26, 27]. However, there are limitations to previous studies using ferrocene‐based sensing techniques, as they:

- were often limited to specific molecular architectures,
- were often limited to the detection of a single analyte,
- were often limited to relatively simple analytical systems, and
- often exhibited poor performance in aqueous or complex matrices [6, 25] where these sensors were intended to operate.

Significant advancements in recent years include the incorporation of ferrocene units into more advanced material platforms, including metal‐organic frameworks (MOFs) [33, 34, 35, 36], nanoparticles [37, 38, 39, 40], conductive polymers [41, 42, 43, 44, 45], and carbon‐based nanomaterials [46, 47, 48, 49]. The addition of the ferrocene unit to these advanced materials significantly enhances the surface area, increases the rate of electron transfer, and allows for increased signal amplification, resulting in better sensitivity, selectivity, and longer‐term operational stability than those found in previously reported ferrocene‐based sensors [50, 51].

In comparison to other prior reports and reviews, which tended to be mainly based upon one particular type of sensing mechanism or a limited number of possible applications, this review is a comparative and systemic review of ferrocene‐based sensors using a wide variety of different materials and sensing modalities; this format allows readers to directly compare the most recent advancements in ferrocene‐based sensors with those of previously developed ferrocene‐based sensors and to understand how the selection of materials and design of the sensor affects its analytical performance [6, 25]. Many of the modern ferrocene‐based platforms also provide the capability to develop ratiometric and dual‐signal sensing schemes for enhancing the precision and sensitivity of detection of multiple analytes in real‐world samples, particularly in both environmental and medical applications [34, 35, 37].

The fact that the analytical applications of ferrocene‐based sensors are currently being examined across many fields (food, environment, medicine, and industry) demonstrates their broad utility and how important it is that this area is explored further. Ferrocene‐based sensors have shown great potential to be used for the detection of several analytes (heavy metals, pesticides, drugs, biomarkers, and mycotoxins) with very low detection limits and high reliability. This shows that ferrocene‐based sensors can help develop new green analytical methods and sustainable technologies. In addition, comparing and evaluating all of these developments against each other and previous research using a common framework highlights the innovation and significance of ferrocene‐based sensors as reliable, versatile, and widely applicable tools for numerous real‐world analytical applications.

The field of molecular sensing has shown significant advancements driven by the development of sensing materials and novel platforms. It has been demonstrated through research utilizing the electrochemistry of ferrocene that there are numerous methods to design and develop highly sensitive molecular sensors capable of detecting a vast number of analytes based on their electrochemical characteristics. There are several categories of materials that may be combined with ferrocene (Table 2), such as A) MOFs that generate a very large surface area and can be engineered to produce specific structures. This makes MOFs an excellent choice for use in conjunction with ferrocene as molecular sensors; B) nanoparticles (NPs) such as gold, silver, and platinum nanoparticles that are chemically modified using ferrocene‐derived compounds to facilitate molecular sensing via their electronic properties; C) polymers containing ferrocene units; D) carbon‐based materials (carbon nanotubes, graphene, and carbon dots) that contain ferrocene derivatives to support molecular sensing applications; and E) ferrocene‐containing molecules as molecular sensors, including molecular sensing in biological/clinical medicine.

**TABLE 2:** Overview of classes of ferrocene materials and their applications in analytical chemistry.

Materials	Formula / Sensor Composition	Analyte	Detection Limit	Method	Application	Ref.
MOFs	l‐lysine‐functionalized Ni–Zn bis(dithiolene) MOF with ferrocene‐modified MWCNTs (multiwalled carbon nanotubes)	Tryptophan (Trp) enantiomers	Enantiomeric selectivity (IL/ID) = 1.866	Electrochemical	Chiral recognition; biomedical relevance	[33]
MOFs	Ferrocene‐functionalized MOF: PEDOT:PSS (poly(3,4‐ethylenedioxythiophene):polystyrene sulfonate)/GO (graphene oxide) composite	C‐reactive protein (CRP); lactate	—	Electrochemical	Dual‐biomarker biosensing	[34]
MOFs	1,1′‐ferrocenedicarboxylic acid (1,1′‐Fc) in Ni‐BDC MOF; Fc‐NH_2_ (ferrocene methylamine) in Ni‐HHTP MOF	Glucose	—	Electrochemical	Enzyme‐free glucose sensing	[34]
MOFs	Nanoparticle/CoFc/MOF hybrid	Hydrogen peroxide (H_2_O_2_)	0.032 µM	Ratiometric electrochemical	In situ biomolecule detection	[35]
MOFs	1,1′‐Fc‐Ni‐BDC	Glucose	5.0 µM (S/N = 3)	Electrochemical	Enzyme‐free glucose biosensor	[36]
NPs	MoS_2_‐Fc‐PdNPs (molybdenum disulfide–ferrocene–palladium nanoparticles) with NGQDs (nitrogen‐doped graphene quantum dots)	Golgi protein 73 (GP73)	0.812 ng mL^−1^; 0.0425 ng mL^−1^	Electrochemical/fluorescence	Hepatocellular carcinoma biomarker detection	[37]
NPs	PVA/AgNPs/Au/Fc‐GOD (polyvinyl alcohol/silver nanoparticles/gold/ferrocene–glucose oxidase)	Glucose	0.038 mM	Electrochemical	Wearable glucose sensor	[38]
NPs	Fc/ZnO/NUNCD/Si (ferrocene/zinc oxide/nitrogen‐incorporated nanodiamond/silicon)	Glucose	1125.18 µA·mM^−1^·cm^−2^	Electrochemical	Nonenzymatic glucose sensing	[39]
NPs	Dimethyl‐Fc electrode with Co–NC–Pd NPs (cobalt‐nitrogen‐doped carbon/palladium nanoparticles)	NADH (nicotinamide adenine dinucleotide, reduced form)	2 µM	Electrochemical	Biomolecular sensing	[40]
Polymers	COF–MIP (covalent organic framework–molecularly imprinted polymer) with Fc crosslinker	Serotonin (5‐HT)	0.03 µM	Ratiometric electrochemical	Neurotransmitter sensing	[41]
Polymers	PF‐b‐PFMMA‐b‐PPEGMA@CD (cyclodextrin–ferrocene block copolymer)	Biomolecular substrates	—	Fluorescence	Drug‐delivery‐related sensing	[42]
Polymers	Fc‐MIP/COF porous carbon	Bisphenol A (BPA)	0.03 µM	Ratiometric electrochemical	Environmental BPA detection	[43]
Polymers	PVA/SA/PEI‐Fc hydrogel (polyvinyl alcohol/sodium alginate/polyethyleneimine‐ferrocene)	H_2_O_2_	—	Electrochemical	ROS (reactive oxygen species) monitoring	[44]
Polymers	GO/LPEI‐Fc (graphene oxide/linear polyethyleneimine‐ferrocene)	Glucose	0.28 mM	Electrochemical	Biofuel‐cell sensor	[45]
Carbon‐based	GC/CNT‐Fc/NiCr_2_O_4_ (glassy carbon/carbon nanotube–ferrocene/nickel chromium oxide)	AM, AT, AS (amlodipine, atorvastatin, acetylsalicylic acid)	0.87–1.26 nM	Electrochemical	Drug analysis in serum	[46]
Carbon‐based	Fc‐lipoic acid modified electrode	Ochratoxin A (OTA)	11 pg mL^−1^	Electrochemical	Food safety analysis	[47]
Carbon‐based	CMWNT‐Fc‐PMo_10_V_2_ (carboxylated MWCNT–ferrocene–polyoxometalate)	Bisphenol A (BPA)	0.035 µM	Electrochemical	BPA detection in milk	[48]
Carbon‐based	Fc‐MWCNT electrode (ferrocene‐functionalized MWCNTs)	Nicotine (NIC)	0.44–4.25 µM	Electrochemical	Nicotine detection	[49]
Fc‐molecule systems	Fc‐biphenyl‐pyridine Schiff base	Ganciclovir	—	Electrochemical	Pharmaceutical sensing	[52]
Fc‐molecule systems	Fc‐BPy (ferrocene–biphenyl pyridine) sensor	Catechol	6.2 µM	Electrochemical	Catechol detection	[52]
Fc‐molecule systems	Ferrocene carboxylic acid	Glucose	—	Electrochemical	Continuous glucose monitoring	[53]
Fc‐molecule systems	CS‐Fc (chitosan–ferrocene hybrid)	Cardiac troponin I (cTnI)	3.70 × 10^−3^ ng mL^−1^	Electrochemical	Acute myocardial infarction biomarker detection	[54]

One of the most important trends in terms of sensitivity among the various material types is the carbon‐based ferrocene‐type platform that demonstrates the lowest detection limits of ochratoxin A at 11 pg/mL and pharmaceuticals at 0.87–1.26 nM. These results reflect the significant conductivity and electrochemically active surface area of these carbon‐based ferrocene platforms [46, 47, 55]. The ferrocene‐nanoparticle systems also exhibit significant sensitivity, especially for protein biomarkers such as GP73, with a limit of 0.0425 ng/ml. These systems benefit from both catalytic and dual‐mode signal amplification [37]. The MOF‐based sensor systems generally fall between others in terms of sensitivity; for example, they have exhibited limits of 0.032 µM for H_2_O_2_ and 5.0 µM for glucose. While the MOF‐based systems possess high porosity that could potentially lead to increased sensitivity, they have limited intrinsic conductivity [35, 36]. In comparison with the MOF and NP‐based sensor systems, the polymer‐based sensor systems typically have a significantly lower level of sensitivity, for example, serotonin (0.03 µM) and glucose (0.28 mM). Finally, the pure ferrocene molecule‐based systems have shown a micromolar‐level sensitivity, as evidenced by a catechol concentration of 6.2 µM [41, 45]. In conclusion, hybrid nanostructured systems that include both carbon‐based materials and NPs are consistently found to provide the greatest analytical sensitivity.

In this review, we explore the diverse applications of ferrocene‐based compounds in molecular sensing, specifically focusing on their integration with different materials to improve their sensing performance.

## 2 Applications

### 2.1 Ferrocene‐Based Molecular Sensors for Environmental Monitoring

Ferrocene compounds have found great utility as molecular sensors in environmental monitoring due to their exceptional ability to sense chemical and physical stimuli and their high reversibility and stability upon redox processes [56, 57, 58, 59]. Therefore, these characteristics make ferrocene compounds ideal candidates for the environmental sensing of trace amounts of pollutants and contaminants [60, 61, 62]. The ferrocene compounds have shown an impressive ability to detect trace amounts of pollutants and contaminants, such as heavy metals [63, 64, 65, 66, 67, 68], organic compounds [69, 70, 71], and VOCs. The ability of ferrocene compounds to detect trace amounts of pollutants and contaminants is mainly attributed to the redox activity of the iron atom present in the ferrocene molecule, which has the ability to respond to even slight changes in the chemical environment.

Additionally, ferrocene compounds have shown a high level of selectivity toward the pollutants and contaminants that they are designed to detect. Therefore, they can accurately distinguish among different types of pollutants and contaminants [50]. Therefore, ferrocene compounds are highly useful for precise and selective environmental monitoring.

There are several ways that ferrocene compounds may be utilized as molecular sensors. One common method is to embed ferrocene compounds within solid‐state materials, such as polymers [51] or membranes [64, 72, 73], to create sensors for detecting and quantifying pollutants and contaminants in the environment. For example, ferrocene‐containing polymers have been used to detect and quantify trace metal contamination in water samples, and ferrocene‐containing membranes have been used to detect and quantify trace levels of VOCs in the air. More recently, researchers have developed MOFs functionalized with ferrocene derivatives as powerful tools for sensing applications in complex environmental matrices [74]. These hybrid materials represent a fusion of the large surface area and tunable porosity of MOFs with the superior redox properties of ferrocene. As a result, these MOF/ferrocene hybrids function as highly selective and sensitive sensors. By incorporating ferrocene units into the framework of MOFs, researchers have created sensors capable of simultaneously concentrating the analyte and detecting it through electrochemistry. Moreover, this concentration capability of the MOF framework provides enhanced sensor performance when dealing with difficult matrices such as tap water, lake water, and complex aerosols from e‐cigarettes. In addition, the porous nature of the MOF structure allows for size‐selective analyte adsorption while the ferrocene groups provide stable and repeatable electrochemical signals. Furthermore, these sensors have shown excellent stability in actual‐use scenarios, with sensors demonstrating consistent performance over many cycles of measurement and excellent resistance to fouling by interferents. Additionally, the synthetic modularity of MOFs offers the opportunity to rationally design ferrocene‐functionalized MOF frameworks targeted at specific analytes or application needs [74].

An additional way that researchers have used ferrocene compounds as molecular sensors is in solution‐based systems, including both electrochemical and optically based sensors. Electrochemical sensors function by detecting and quantifying contaminants and pollutants by measuring the changes in the electrical properties of the ferrocene compound as it reacts with the target contaminant. On the other hand, optically based sensors function by utilizing the changes in the absorbance or fluorescence of the ferrocene compound as it reacts with the target contaminant to detect and quantify the contaminant [75].

Overall, ferrocene compounds have been highly successful as molecular sensors for environmental monitoring because of their high sensitivity, selectivity, and versatility. Ferrocene compounds will likely remain important contributors to environmental monitoring efforts in the years ahead due to their potential to greatly enhance the accuracy and efficiency of these efforts.

#### 2.1.1 Ferrocene‐Based Compounds to Detect Heavy Metals

**FIGURE 1:**
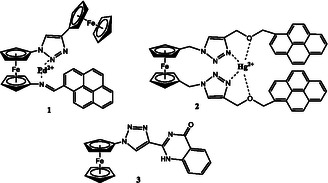
Triazolyl‐appended ferrocene sensors for Hg^2+^ (**2** and **3**) and Pd^2+^ (**1**).

Ferrocene scaffolds have demonstrated exceptional utility as molecular recognition platforms, with researchers developing sophisticated receptor architectures including mono‐ and di‐substituted derivatives, macrocyclic and acyclic frameworks, and both charged and neutral recognition motifs [56, 57, 58]. This structural diversity enables the precise tuning of binding affinity and selectivity for specific target analytes. Studies have reported their potential in recognizing cations, anions, and ion pairs [56, 59, 60, 61]. Heterocycles, such as oxazoline, triazole, and imidazole, have been shown to be highly efficient detection units, particularly for cations [62, 63]. Ferrocenyl‐triazole derivatives have been developed as multiple signaling molecular chemosensors for various ions, including Fe^2+^/Fe^3+^, Hg^2+^, and Pb^2+^ (Figure 1) [25, 64, 65, 66]. In ferrocenyl‐triazole compounds, cation linking at an adjacent receptor unit provokes a positive shift in the redox potential of the Fc/Fc^+^ redox couple, modulating on/off the complexation ability of the triazole ligand by adjusting the electrochemical potential response. The magnitude of the electrochemical shift (Δ*E*_1/2_) upon complexation serves as a quantitative measure of the perturbation experienced by the redox center due to its interaction with the receptor unit [50, 51, 59, 67, 69, 70, 71].

Several ferrocene‐triazolyl complexes were evaluated for their ability to bind to cations. Notably, compound **1** exhibited a high degree of selectivity for Pd^2+^ over other elements, with a 1:1 binding ratio between the ligand and metal, a favorable detection limit of 7.2 x 10^−6^ M, and a color change upon binding [72]. In addition, Hg^2+^ was found to be the most detectable and selective cation within the framework of compound **2**, with a 2 ppb detection limit and a 1:1 binding ratio [73], or a 1:2 binding ratio when compound **3** was utilized (as depicted in Figure 1) [64]. Electrochemical analysis demonstrated that the behavior of compound **2** was distinguishable from that of other cations in certain ferrocene‐triazolyl models, as no perturbations were observed in the cyclic voltammetry with several metal ions, except for Hg^2+^ ion [74, 75]. Therefore, it is necessary to examine a variety of cations, including Ca^2+^, Mg^2+^, Cr^3+^, Al^3+^, Zn^2+^, Fe^3+^, Ni^2+^, Fe^2+^, Co^2+^, Cu^2+^, Cd^2+^, Pb^2+^, and Hg^2+^, to explore the detectable elements using the complexes described in this study.

Despite the encouraging outcomes observed in prior studies investigating ferrocenyl‐substituted molecular sensors, such as their high selectivity and detection techniques [76], further research is required to devise efficient ferrocene‐based systems that are effective in aqueous media, exhibit higher binding constants or lower detection limits, and demonstrate enhanced selectivity toward physiological components, particularly zinc, as current cation recognition has predominantly been conducted in organic solvents, presenting significant challenges for biological applications [56]. Additionally, limited studies have explored ferrocene derivatives for mercury(II) detection in aqueous environments, with research focusing on developing chromogenic and electrochemical detection methods [59].

Previous studies have demonstrated the effectiveness of ferrocene‐based materials for electrochemical detection of specific heavy metals in aqueous solutions. A ferrocene functionalized graphene oxide nanocomposite was developed to detect lead ions via electrochemistry. The researchers achieved a lead ion detection limit of 0.168 micrograms per liter [77]. Additionally, a ferrocene appended chalcone (FAC) compound, which has been utilized as both an electrochemical and chromogenic sensor, was synthesized to selectively quantify copper(II) ions. The researchers reported detection limits for the FAC compound based on the detection method with 1.78 x 10^−7^ M (electrochemical) or 6.00 x 10^−7^ M (chromogenic) [78, 79]. The high selectivity and sensitivity for heavy metals exhibited by these ferrocene‐functionalized materials indicate potential utility in developing sensors for lead monitoring using the graphene oxide nanocomposite and for copper analysis using the chalcone‐based sensor. Although both studies demonstrated the ability to utilize ferrocene as a redox‐active component in sensing platforms for heavy metals, they achieved this by optimizing the materials for use in detecting a single specific metal rather than a range of heavy metals. Electron transfer in clay minerals with iron(III) was investigated using ferrocenyl surfactants. Ferrocenyl surfactants, having different lengths of hydrocarbon chains, were examined to determine the level at which these surfactants could physically interact with reactive sites on clays, as well as the thermodynamic favorability of the redox reaction occurring between the surfactant and iron(III).

Longer‐chain surfactants facilitated electron transfer due to better access and favorable conditions, as indicated by spectroscopy and visible color changes. The shorter‐chain surfactant was not involved in electron transfer due to its rigid structure and high oxidation potential. The study examined the feasibility and limitations of these surfactants as template compounds for electron transfer studies [80]. Overall, these recent environmental studies demonstrate the potential of ferrocene compounds for the detection of trace levels of heavy metals. Ferrocene compounds have been proven to be highly selective and sensitive for the detection of heavy metals in different media consisting of water and soil. In addition, they have the potential to be incorporated into portable and handheld devices for heavy metal detection, which could be highly effective for on‐site detection applications.

#### 2.1.2 Ferrocene‐Based Compounds to Detect Pesticides

A ferrocene nanoporous organic polymer (Fc‐NOP), incorporating aromatic cyclopentadienyl unit moieties, has been found to give the resultant nanoporous organic polymer enhanced features such as catalytic activity [81] and improved gas adsorption [82, 83, 84]. Due to the presence of the cyclopentadienyl ion in ferrocene, it has a good affinity for aromatic systems through π‐π interaction. Fc‐NOP, which exhibits good physicochemical stability, high reusability, and efficient adsorption capacity for chlorophenols (CPs), was used as a solid‐phase extraction (SPE) adsorbent to remove some trace CPs, such as 2‐chlorophenol, 2,3‐dichlorophenol, 4‐chlorophenol, and 2,4‐dichlorophenol [85].

Moreover, ferrocene‐based dendrimers (FcDr) have illustrated effective sensing molecules and molecular receptors due to their ferrocene moiety, which is fashioned to be an electrochemical signaling unit integrating into bonding reactions via electrostatic attraction and chemical bonding [86, 87, 88]. As a result, they have been frequently employed as amperometric sensor mediators or as coatings to change electrode surfaces [61, 89]. Recently reduced graphene oxide nanosheets and gold NPs covalently linked to ferrocene‐terminated dendrimers were used as an electrochemical signal amplifier for ultra‐sensitive detection of pesticides such as dichlorvos (2,2‐dichlorovinyl dimethyl phosphate) [90].

**FIGURE 2:**
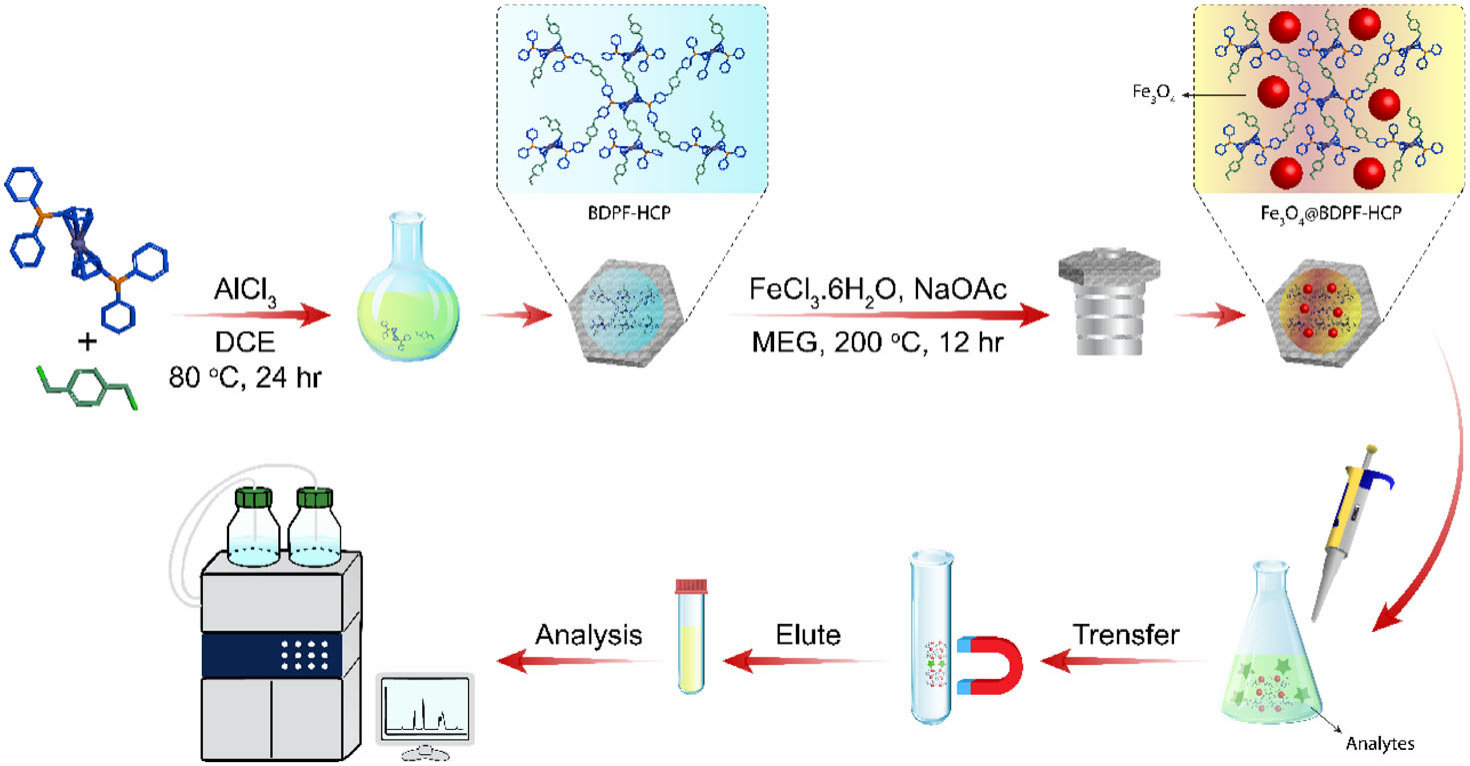
Schematic diagram of the preparation and magnetic solid‐phase extraction (MSPE) process of Fe_3_O_4_@BDPF‐HCP.

Very harmful pesticides such as CPs can be detected using magnetic hyper crosslinked polymers (HCPs) Fe_3_O_4_@BDPF‐HCP, which are generated via Friedel–Crafts crosslinking of a 1,1′‐bis(diphenylphosphino)ferrocene (BDPF) monomer with various external crosslinkers, followed by a modification with Fe_3_O_4_ NPs. This recently developed ferrocene‐based polymer acts as a magnetic solid‐phase extraction adsorbent for some chlorophenols [91]. Figure 2 illustrates the preparation steps of the Fe_3_O_4_@BDPF‐HCP material and its application in magnetic solid‐phase extraction. The schematic emphasizes the dual function of the system, where the ferrocene moieties facilitate analyte interaction while the magnetic core enables rapid and efficient separation, contributing to improved extraction performance.

Overall, while ferrocene‐functionalized MOFs show strong analytical performance, their practical application is often limited by issues of stability and scalability.

### 2.2 Ferrocene Compounds as Biosensors for Medical Diagnosis

Biosensors represent sophisticated analytical instruments that leverage biomolecular recognition mechanisms to achieve highly selective detection and quantification. Their widespread adoption stems from the exceptional combination of analytical specificity, sensitivity, and cost‐effectiveness they provide. Among various biosensor platforms, electrochemical systems have emerged as particularly attractive for biomolecule detection, offering operational simplicity, economic viability, and outstanding analytical performance [92, 93, 94]. The basic structure of these sensors consists of an electrode that is supplied with a recognition molecule specific to the biological marker of interest.

#### 2.2.1 Ferrocene‐Based Compounds to Detect Biomarkers in Biological Fluids

Biomarkers binding to the recognition element generate measurable changes in local electrochemical potential or current, forming the basis for quantitative detection. Since most clinically relevant biomarkers lack inherent electrochemical activity, redox mediators serve as essential signal transduction elements, enabling sensitive electrochemical detection of otherwise electrochemically silent biomolecules [95, 96, 97, 98, 99]. A ferrocene unit plays a significant role in electrochemical sensing and is considered a powerful molecular sensor by facilitating the flow of electrons and ions to promote conductivity. In light of this, Fc can be utilized as a label or an electrochemical mediator due to its conductivity.

Recent advances in biosensor technology have highlighted the potential of ferrocenyl compounds as superior alternatives to conventional redox labels such as methylene blue [100]. A groundbreaking study published in December 2024 demonstrated that ferrocenyl compounds incorporating crosslinking functional groups exhibit enhanced stability and improved electrochemical performance compared to traditional redox mediators [101]. These novel compounds address critical limitations of existing redox labels, including susceptibility to degradation and limited operational stability under physiological conditions. The crosslinking capability of these ferrocenyl derivatives enables more robust immobilization on electrode surfaces, resulting in enhanced sensor longevity and reproducibility. Furthermore, the tunable redox potential of ferrocene derivatives allows for optimization of signal‐to‐noise ratios in complex biological matrices, making them particularly attractive for point‐of‐care diagnostic applications where reliability and stability are paramount [101].

**FIGURE 3:**
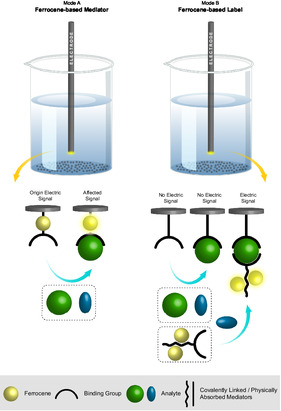
Schematic explanation of Fc‐based performance.

One of the effective ways to improve electron transfer during sensing is the attachment of Fc‐based mediators to the surface of electrodes by covalent bonding or deposition. A blank sample has some electric signal A1, which changes to A2 upon binding of the analyte with the binding group of the sensor. In contrast, the label‐based sensor does not produce any output signals when the analyte interacts with the surface‐bound binding groups; however, it provides anchoring sites for Fc‐based labels. Only after the coupling of such conductive labels based on Fc (B3) do electrochemical signals appear [25]. The schematic comparison of ferrocene‐labeled versus label‐free sensing is provided in Figure 3. As illustrated with the inclusion of ferrocene as an active mediator that can be oxidized/reduced, the efficiency of the signal generated will increase relative to label‐free sensing approaches due to the mediation role ferrocene plays. Amplified signals are produced by ferrocene‐tagged systems as a result of signal enhancement through analyte binding; this results in greater sensitivity for ferrocene‐tagged systems than those using label‐free approaches.

By surface modification, appropriate electrodes have been fabricated. There are three main categories of sensors: membrane‐ or film‐type sensor, sandwich‐type sensor, and shift‐type sensor. It has so far been exhibited that Fc‐containing moieties combined with sensor substrates tend to improve ion transport, as was observed in a film‐type glutamate sensor made of Fc derivatives and PVC [96]. Other cases involved the identification of hypoxanthine in fish muscles using a polypyrrole film immobilized with Fc carboxylic acid and xanthine oxidase [102].

Moreover, for ion sensing, a membrane‐type sensor containing an Fc‐PVC hybrid system was utilized [103]. A sensor incorporating an Fc‐peptide nanowire‐antibody (Fc‐PNW‐Ab_1_) framework and featuring tumor necrosis factor α (TNF‐α) detection with an Ab_2_‐gold nanorod‐glucose oxidase label demonstrates excellent selectivity and sensitivity as a sandwich‐type biosensor for TNF‐α protein biomarker detection. The dual signal amplification strategy employs ferrocene moieties on the peptide nanowire as mediators for glucose oxidase to catalyze the glucose reaction, generating an electrochemical response proportional to TNF‐α concentration. In general, catalytic oxidation in the presence of Fc generates a response with the addition of glucose, resulting in an increase in current corresponding to higher amounts of the target analyte present [99].

Besides film‐type and sandwich‐type sensors, assistant material, including NPs that provide a compromise between biocompatibility and conductivity for Fc‐based sensors, such as magnetic NPs and Au NPs, has been explored. Notable examples of Fc‐based electrochemical sensors have been published using this type of assistant material [25, 99, 101].

**FIGURE 4:**
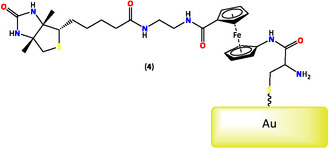
1′‐Fmoc‐amino‐ferrocene‐1‐carboxylic acid molecule as promising biosensors.

Targeting large biomolecular targets can be accomplished by implanting large antibodies or immunoglobulins into a ferrocene‐based compound on an electrochemical surface [104, 105]. Researchers prepared the orthogonally substituted 1′‐Fmoc‐amino‐ferrocene‐1‐carboxylic acid molecule (4), a bioconjugate ferrocene system with a receptor component (biotin), an electrochemical redox system (ferrocene), and an immobilizing‐linker component (cysteine), all of which were immobilized on a gold surface as promising biosensors, as seen in Figure 4 [106].

#### 2.2.2 Ferrocene‐Based Compounds as Sensors for Protein

**FIGURE 5:**
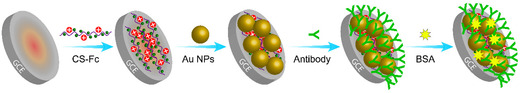
The stepwise fabrication process of the immunosensor BSA (bovine serum albumin).

A label‐free amperometric immunosensor for hepatitis B surface antigen (HBsAg) has been produced using biocompatible conductive redox chitosan‐ferrocene/gold (CS‐Fc/Au) NPs. The Fc moiety (which functions as a mediator) was linked to the long‐chain CS to generate the unique redox‐active hybrid material (CS‐Fc), which was then electrodeposited onto the surface of a glassy carbon electrode (GCE). The stepwise fabrication of the immunosensor presented in Figure 5 offers a clear overview of the surface modification process and biomolecule immobilization. This schematic helps clarify how each functional layer contributes to selective target recognition and overall sensor performance. As demonstrated in Figure 5, the amino groups of CS worked as active sites for the immobilization with AuNPs, providing an interface for the immobilization of hepatitis B surface antibody (HBsAb). For HBsAg, this biosensor demonstrated high sensitivity and a low detection limit [100, 107, 108, 109, 110, 111].

**FIGURE 6:**
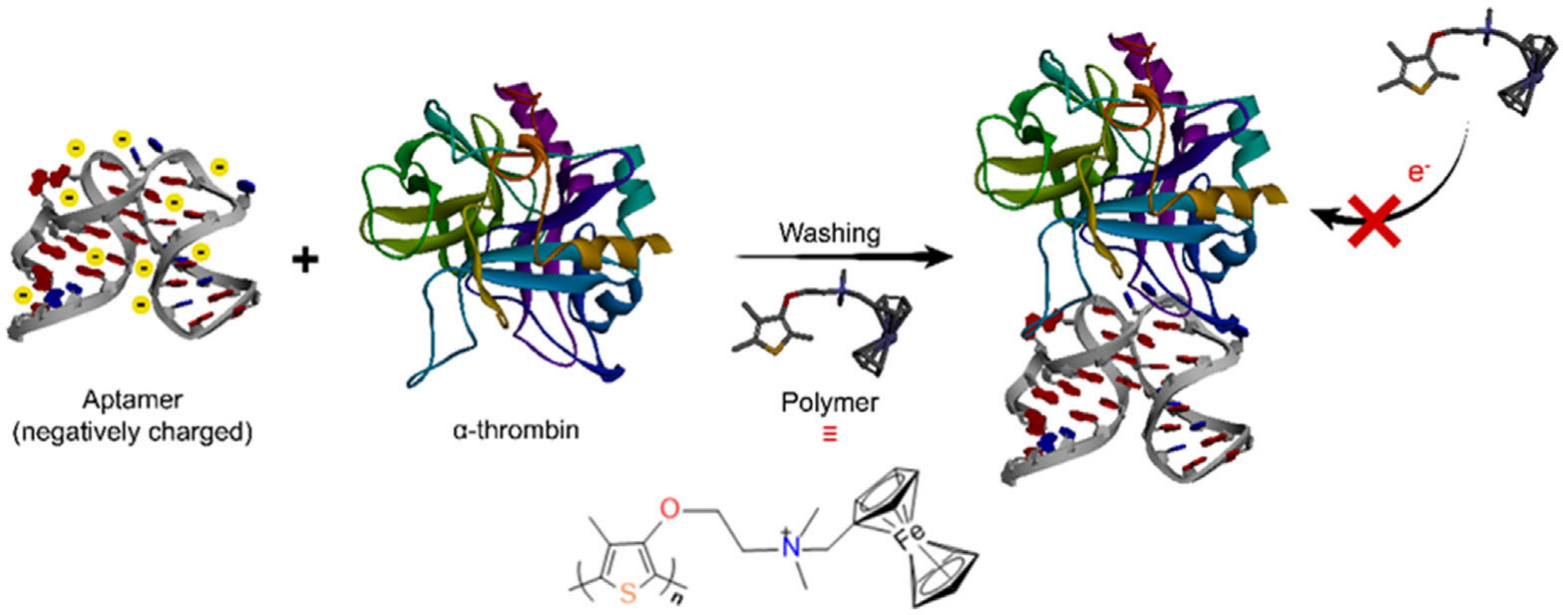
Illustrative diagram for the detection of α‐thrombin.

In another application, human α‐thrombin was detected using polymer 9. This was achieved using a gold electrode surface that was functionalized with a human α‐thrombin aptamer (an oligonucleotide). After modification, the gold electrode was submerged in analytical human α‐thrombin solution and properly rinsed. Then, the modified electrode was investigated using square‐wave voltammetry in a polymer solution with and without α‐thrombin. It was noticed that the electrode without α‐thrombin has a higher current peak than the electrode with α‐thrombin (Figure 6) because of the presence of the negatively charged aptamer. This is explained by the fact that in the absence of α‐thrombin on the electrode, the cationic polymer readily accessed the anionic surface of the electrode and gave a large current peak [112].

#### 2.2.3 Ferrocene‐Based Compounds as Sensors for Enzymes

**FIGURE 7:**
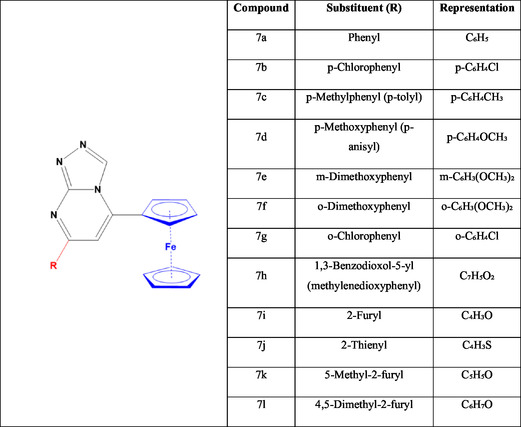
Chemical structure of synthesized 5‐ferrocenyl triazolopyrimidine derivatives **7(a‐l)**.

A hybrid system of ferrocene‐based triazolopyrimidines (**6a‐o**) has the ability to modulate pyruvate kinase M2 (PKM2), a glycolytic enzyme that converts phosphoenolpyruvate (PEP) into pyruvate and is highly expressed in oral, lung, breast, and colorectal malignancies (Figure 7). The mechanism of such hybrid systems contains a heterocyclic polar part as well as functional groups that interact with the amino acids of cancer target proteins. On the other hand, the ferrocenyl moiety in a cylindrical orientation may fit well in the space of the target protein's hydrophobic pockets and may act as a redox sensor. In addition, the ferrocene head may preferentially drive the molecules toward hypoxic conditions, while the heterocyclic portion may interact with the target's essential residues, resulting in anticancer activity [113].

**FIGURE 8:**
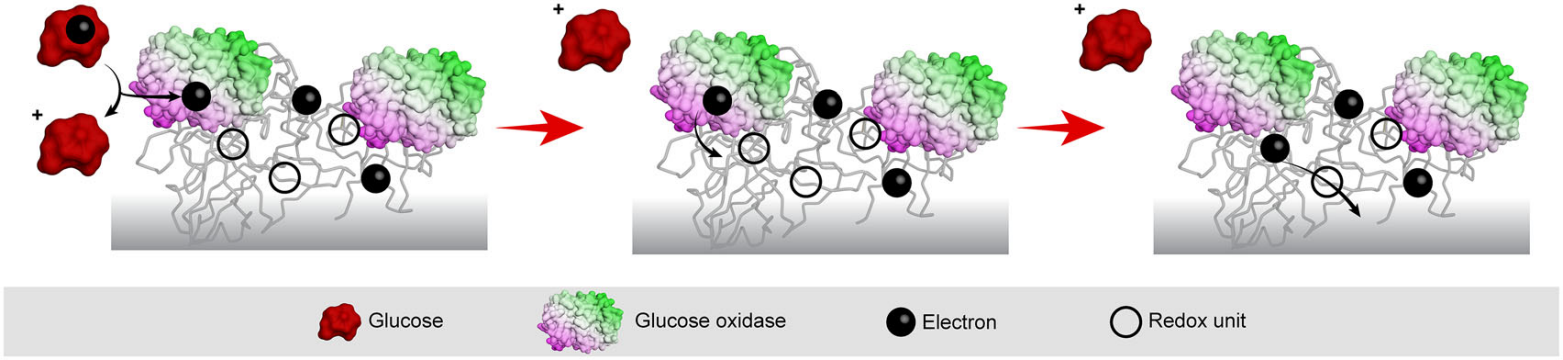
Redox hydrogel with ferrocene polymer to detect glucose.

In another example, a photochemical technique was used to create functional, enzyme‐containing redox hydrogels independent of specific functional groups in the ferrocene polymer backbone. The redox polymers with photo‐reactive benzophenone units inserted on the electrode surface are then crosslinked by UV irradiation. If there are active biocatalyst molecules in the deposited film, they are covalently linked within or physically entrapped inside the network. This simple and adaptable method is based purely on the reaction between benzophenone and nearby CH groups, so it can be used to crosslink redox polymers with a variety of functionalities and compositions (Figure 8) [114].

**FIGURE 9:**
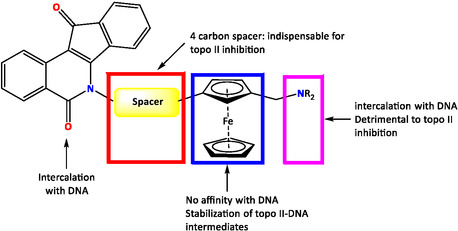
Structure–activity relationships of ferrocenyl derivatives.

The ferrocene‐based compounds were also utilized as inhibitors of topoisomerase II (Topo II). Topoisomerases I and II are indispensable enzymes in the processes of DNA replication, transcription, and recombination. Researchers concentrated on the synthesis of indeno[1,2c]isoquinolines, including the incorporation of ferrocenyl units, and tested their inhibition activity against topoisomerase. Supercoiled plasmid DNA was treated with the topoisomerase enzyme in the presence of test compounds. The results showed that the primary amine of N6 lactam 196 containing the ferrocenyl moiety plays an important role in the inhibition of Topo II and possesses stronger poisoning action compared to the compound lacking the ferrocenyl group, which had no effect. The structure–activity relationship indicated that the length of the N6 side chain influences Topo II inhibition; the compound bearing a four‐methylene spacer had more activity compared to the three‐methylene spacer one (Figure 9) [115].

### 2.3 Ferrocene Compounds in Industrial Monitoring

#### 2.3.1 Ferrocene‐Based Compounds as Sensors for Volatile Organic Compounds (VOCs)

A material called ferrocene‐tetrazine porous organic polymer (FcTz‐POP), which has ferrocene building blocks, was synthesized along with a reference material called biphenyl‐tetrazine porous organic polymer (BpTz‐POP), which does not have ferrocene. FcTz‐POP was found to capture iodine much more effectively than BpTz‐POP, with a capturing capacity of up to 396 wt% at 348 K and atmospheric pressure. This is mainly due to the cyclopentadienyl group in ferrocene, which has a stronger interaction with iodine than the benzene ring and the Fe^2+^ on ferrocene, which may bind negatively charged polyiodides through electrostatic interactions. Additionally, FcTz‐POP exhibited a fast adsorption rate for iodine vapor in the first 7 h due to the combined effects of ferrocene units, heteroatoms (N), and a conjugated π electron‐rich aromatic system. These findings suggest that incorporating functional groups with a high affinity for iodine into the material is an effective strategy for achieving high iodine loading capacities for amorphous materials [116].

#### 2.3.2 Dual Detection Systems

The integration of ferrocene derivatives into dual‐mode detection systems represents a significant advancement in analytical chemistry, offering complementary optical and electrochemical readouts for enhanced reliability and accuracy [117]. Recent developments have focused on ferrocene‐chalcone hybrid probes that enable simultaneous fluorescence and electrochemical detection of target analytes such as carbon monoxide [117]. These dual‐mode systems provide several advantages over single‐detection methods, including built‐in validation through orthogonal measurement principles and improved signal reliability in complex matrices. The ferrocene component provides a stable electrochemical signal through its reversible redox behavior, while the chalcone moiety contributes fluorescence properties that can be modulated by analyte binding [117].

This approach is particularly valuable for safety‐critical applications such as carbon monoxide detection, where false negatives could have serious consequences. The optical component also enables remote sensing capabilities and visual confirmation of detection events, making these systems suitable for both laboratory and field applications. The excellent optical performance combined with robust electrochemical response positions make these dual‐mode ferrocene‐based sensors the next‐generation analytical tools [117].

### 2.4 Ferrocene Compounds in Food Safety

The notable electrochemical characteristics of ferrocene compounds, such as impressive stability and fast electron transfer rates, have made them valuable in a diversity of applications. Ferrocene compounds have been used in food safety, and this use has recently attracted strong interest [115]. For the aim of detecting various contaminants and pathogens in food, such as heavy metals, insecticides, mycotoxins, fungi, and bacteria, FBSs were designed.

These sensors have several advantages over conventional analytical approaches, including low detection thresholds, high sensitivity, and fast reaction times [116, 118]. The identification of heavy metals is one of the most promising uses of FBSs in food safety. Because they may accumulate in food sources and are harmful to people, heavy metals pose a significant health threat. Lead, mercury, and cadmium are examples of heavy metals that have been detected in food samples using FBSs. When the heavy metal ion exists, these sensors recognize changes in the ferrocene compound's electrochemical characteristics [119]. Ferrocene compounds have also confirmed potential in the detection of mycotoxins, which are toxic substances made by fungi that can pollute food. FBSs have high sensitivity and specificity for the detection of mycotoxins, including ochratoxin and aflatoxin [120]. Moreover, Salmonella and E. coli are two examples of bacterium‐induced infections that can be detected in food when using FBSs. When bacterial cells or their metabolites are found, these sensors identify variations in the electrochemical properties of the ferrocene molecule [121].

#### 2.4.1 Ferrocene‐Based Compounds as Sensors for Determination of Pesticide

By creating the novel chemical FT‐Pr, which contains electroactive ferrocene groups and electropolymerizable thiophene, a novel hybrid nanosensor, the ferrocene‐thiophene@carbon nanotube (FT@CNT) nanosensor, was synthesized. Azide‐functionalized carbon nanotubes and FT‐Pr were fused via the click reaction. The hybrid FT@CNT nanosensor's surface area and conductivity were boosted by the integration of thiophene and carbon nanotubes, while its electron transport and electroactivity were developed by the addition of ferrocene. Utilizing the differential pulse voltammetry (DPV) approach, this unique nanosensor was used to measure the amounts of parathion and chlorantraniliprole in food samples (apple and tomato) and soil. The FT@CNT nanosensor demonstrated high selectivity, sensitivity, and stability for the detection of both analytes in real samples. This combination of CNT, thiophene, and ferrocene provided a superior nanosensor system [118].

**FIGURE 10:**
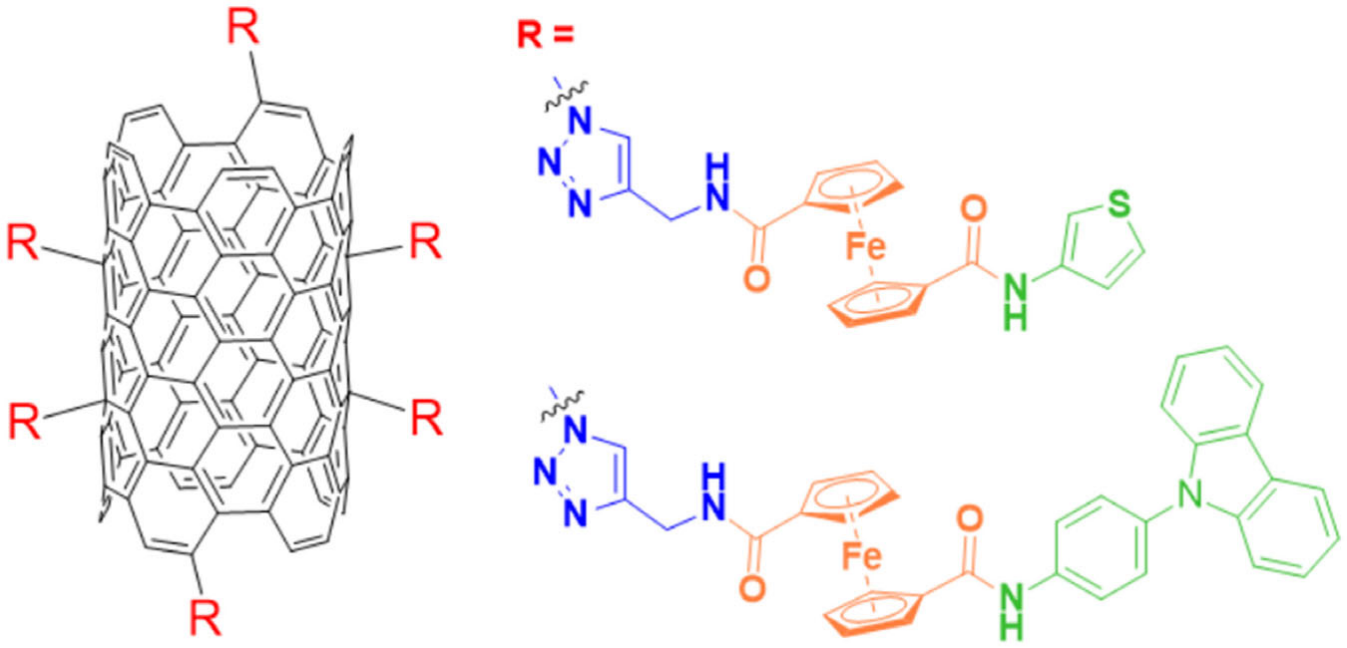
Carbon nanotubes functionalized via triazole linkers with ferrocene‐based ligands bearing (**R**) either 2‐thienylmethylamine or an N‐(piperidin‐4‐yl) carbazole moiety to enhance electrochemical sensing performance.

The surface of carbon nanotubes (CNTs) was functionalized via a click reaction with a novel ferrocene‐carbazole derivative, combining ferrocene as an electroactive moiety and carbazole as an electropolymerizable unit (Figure 10). This approach yielded a hybrid carbon nanotube material (CFC) exhibiting excellent chemical stability and enhanced electrochemical properties. The modified CNTs demonstrated high sensitivity and selectivity in the simultaneous voltametric detection of the pesticide's chlorantraniliprole and spinosad. This hybrid nanosensor offers a promising platform for rapid, sensitive, and reliable pesticide monitoring in environmental and agricultural samples [119].

A group of researchers synthesized a set of podophyllotoxin esters that contained ferrocene scaffolds. The study found that some compounds exhibited the highest insecticidal activity against Mythimna separata, while the others showed the most potential acaricidal activity against *Tetranychus cinnabarinus*. The study highlighted that a 6,7‐methylenedioxy group is essential for insecticidal activity, while 6,7‐dimethoxy substitution enhances acaricidal effects; halogenation of the E‐ring and ferrocene addition at C‐4 also increased potency. These findings support further development of podophyllotoxin derivatives as eco‐friendly botanical pesticides [120].

#### 2.4.2 Ferrocene‐Based Compounds as Sensors for Determination of Mycotoxins

A new electrochemical aptamer sensor capable of simultaneously detecting two mycotoxins, Ochratoxin A (OTA) and Aflatoxin B1 (AFB1), was developed, and its performance was evaluated. The sensing substrate was a GCE modified with gold NPs‐carbon nanodots (AuNPs‐CNDs). OTA and AFB1 were detected using two different electroactive bioconjugates, which are based on a MOF named HKUST‐1 (Hong Kong University of Science and Technology‐1), which is synthesized by copper nodes and complementary DNA for OTA aptamer (cDNA1) and for AFB1 (cDNA2): hemin@HKUST1/cDNA1 and ferrocene@HKUST‐1/cDNA2. By employing the differential pulse voltammetry technique to measure the peak currents of the electroactive bioconjugates, the concentration of the mycotoxins could be obtained. At concentrations between 1.0 x 10^−2^ and 100.0 ng mL^−1^, the aptasensor was able to identify both mycotoxins. This dual‐signal electrochemical aptasensor shows encouraging potential for the determination of many mycotoxins at the same time in diverse samples [121]. For the highly sensitive analysis of OTA, a unique electrochemical sensor based on an aptamer was synthesized. Five single‐stranded DNA strands were utilized by the sensor to manage stepwise fabrication of dual AuNP couplings, formulating two AuNP conjugates with similar sizes but unique oligonucleotide modifications. The two conjugates advanced Fc electroconductivity and load abundance. The aptamer's recognition of the OTA caused the first AuNP conjugate to be removed from the electrode surface, producing the CP2 inside the single‐strand configuration. As a result, the signal was proportionally equivalent to the OTA. This concentration may enhance a good sensing platform for OTA's trace‐level biochemical research [122].

#### 2.4.3 Ferrocene‐Based Compounds as Sensors for Determination of Heavy Metal

Fc‐NH_2_‐UiO‐66 (University of Oslo‐66 MOF), a ferrocene‐based molecule functionalized with carboxylic acid, is an example of an MOF that may be used as a sensor for heavy metal ions [123]. MOFs are pore‐filled, crystalline structures made of metal ions or clusters joined by organic ligands. They feature large surface areas, variable pore sizes and shapes, and different functions. A MOF with ferrocene and amino groups on the ligands is Fc‐NH_2_‐UiO‐66. While the ferrocene groups can show electrochemical signals, the amino groups can serve as sites for heavy metal ions to adsorb. By calculating the peak current ratio of ferrocene and heavy metal ions, Fc‐NH_2_‐UiO‐66 may be used as a ratiometric electrochemical sensor for the parallel determination of many heavy metal ions, such as Cd^2+^, Pb^2+^, and Cu^2+^. The advantages of this approach are high selectivity, repeatability, dependability, and sensitivity [123].

A ferrocene‐functionalized aptamer is another example of a ferrocene‐based molecule used as a heavy metal ion sensor [124]. Short single‐stranded DNA or RNA molecules known as aptamers have a high affinity and selectivity for binding to specific targets. Aptamers that have ferrocene groups attached to their endpoints or internal locations are termed “ferrocene‐functionalized aptamers.” For signal transmission, the ferrocene groups can act as optical or electrochemical labels. By monitoring the conformational changes or interactions that occur between the aptamers and the metal ions, ferrocene‐functionalized aptamers can be utilized as sensors for heavy metal ions. For example, the formation of a ferrocene‐functionalized aptamer sensor for Pb^2+^ assumed the G‐quadruplex structure's preparation in the presence of Pb^2+^, which led to an amplified ferrocene electrochemical signal [124].

### 2.5 Advanced Multiferrocene Architectures

The development of multiferrocene‐based ligands represents a significant advancement in molecular sensor design, as comprehensively reviewed previously. These multiferrocenyl‐containing systems offer unprecedented opportunities for signal amplification and enhanced selectivity through cooperative binding effects [125]. Unlike single ferrocene units, multiferrocene architecture enables multiple redox events per binding interaction, dramatically improving detection sensitivity. The synthetic strategies for these complex ligands have evolved to include controlled polymerization techniques, dendrimer synthesis, and metal‐organic framework incorporation, allowing precise control over ferrocene spacing and orientation [125]. In sensing applications, these multiferrocene systems demonstrate superior performance in detecting low‐abundance targets, with some configurations achieving detection limits several orders of magnitude lower than their mono‐ferrocene counterparts. The ability to fine‐tune electronic communication between multiple ferrocene centers also enables the development of ratiometric sensors that provide built‐in calibration and improved accuracy in complex sample matrices [125].

While many different types of ferrocene‐based sensing systems have been developed, there is evidence that their performance is heavily dependent on the type of material they are supported by. For example, ferrocene‐MOF‐based platforms typically exhibit exceptionally high sensitivity and very low detection limits. These enhanced properties can be explained by the large surface area of the MOF material, its ability to be structured in a variety of ways to provide an optimal binding site for the analyte, and its potential to concentrate the analyte close to the redox‐active center of the ferrocene sensor [33, 34, 35, 36]. On the other hand, while the majority of MOFs are capable of achieving the sensitivity levels mentioned earlier, several reports indicate that some MOFs may degrade over time due to exposure to highly aqueous or acidic solutions, which could limit their practical use in the long term [34, 37]. In contrast, ferrocene‐polymer systems tend to be more mechanically flexible and therefore easier to process into wearable sensors for measurements in samples taken from real‐world environments. Furthermore, they tend to demonstrate higher long‐term operational stability when compared to the more rigid MOF‐based systems, although at the cost of potentially slightly lower sensitivity [41, 42, 43, 44, 45]. Finally, ferrocene‐modified sensors using carbon‐based supports such as carbon nanotubes and graphene are able to capitalize on the excellent electrical conductivity and very fast electron‐transfer rates associated with these materials, both of which contribute to high‐quality and rapid sensor signals [46, 47, 48, 49]. However, achieving both reliable surface modification on carbon‐based materials and obtaining high selectivity in complex sample matrices continues to represent challenges for the development of sensors utilizing these materials [47, 48]. Ultimately, these results suggest that no single ferrocene‐based system is universally better than all the others. Rather, the most suitable ferrocene‐based system is one whose characteristics are optimized for the specific sensing application, desired analytical performance, and operating environment.

## 3 Challenges and Future Perspectives

### 3.1 Current Challenges and Limitations

Although the sensing properties of ferrocene‐based sensors are quite impressive, they face several challenges that should be overcome for them to be successfully translated into and used in clinical and commercial settings. The challenges include basic chemical stability issues with the sensor materials, complicated interactions with matrix systems (the system being analyzed), and scaling‐up issues that relate to translating small‐scale laboratory prototype sensors into reliable analytical instruments [79].

#### 3.1.1 Stability and Degradation Challenges

Long‐term operational stability is likely the largest obstacle to implementing ferrocene‐based sensors in practice. Ferrocene derivatives are prone to chemical breakdown (structural degradation) when exposed to physiological conditions. This creates a substantial barrier to achieving long‐term sensor performance. Research has shown that ferrocenium ion undergoes a specific type of chemical reaction known as a molecular displacement reaction when it comes into contact with nucleophiles such as chloride ions and other physiological species. As a result, the ferrocenium ion decomposes and is no longer capable of functioning as a sensor, which results in a loss of the ability to provide a stable sensor response over time [126].

Stability issues are exacerbated for ferrocene‐functionalized MOFs due to their sensitivity to both thermal and electrochemical stress. The combination of these stresses will cause the MOF to collapse and leach the active components. The structural integrity of MOF‐based ferrocene systems is maintained until they reach approximately 200°C, at which point the linkers begin to decompose and break down at approximately 230°C [127]. Even more concerning is that prolonged electrochemical exposure will result in the leaching of the ferrocene linkers, and research has documented that MOFs can lose up to 1% of their mass in 2 h of continuous electrochemical exposure [127]. These degradation processes negatively affect the reliability of the sensor and thus require frequent calibration and/or replacement.

Additionally, aptamers that are part of a ferrocene‐based sensor may experience instability due to several different modes of degradation. Electrode defects induced by surface treatments, solubilization of the monolayer at high temperatures, and biochemical degradation of the aptamer itself, as well as progressive biofilm formation, all act to degrade sensor performance over time [128]. In biological environments, such as physiological matrices, these factors can be exacerbated by the presence of proteins and enzymes that rapidly lead to a loss of both precision and operational life for the sensor.

#### 3.1.2 Matrix Effects and Analytical Interference

Matrix effects caused by biological matrices are a major challenge to the use of FBSs for analytical purposes, especially for clinical diagnostics. Biological matrixes create significant complexity within the clinical sample that results in the degradation of biosensor analytical performance; thus, many biosensors have difficulty transitioning from buffer solutions to actual clinical samples [129]. Ferrocene's high positive redox potential (+0.4V vs. SCE) can cause nonspecific adsorption of proteins onto electrodes, resulting in interference on the electrode surface, which will degrade both the selectivity and accuracy of the sensor's analytical response [126].

The complexity of analyzing biological fluids is compounded by the presence of interfering electroactive substances, which create overlapping electrochemical signals and confuse accurate analyte quantification. Commonly found ascorbic acid, uric acid, and many pharmaceutical compounds can also be detected electrochemically and thereby interfere with the ferrocene‐mediated detection mechanism(s). Physiologically occurring electrolytes can also cause changes in the electrochemistry of ferrocene derivatives by causing signal suppression and/or a shift in potential that negatively impacts quantitative precision.

#### 3.1.3 Commercial Viability and Manufacturing Scalability

Manufacturing scalable, affordable, and reliable devices from laboratory prototypes is an area of challenge for commercialization. Lithography, a fabrication technique used in large‐scale biosensor development, has the potential to facilitate large‐scale fabrication of ferrocene‐based biosensors; however, high precision (required for ferrocene‐based biosensors) at low cost is a major barrier to its successful use [130]. As a result, most current fabrication methods are limited by the requirement of clean‐room‐type facilities or expensive special‐purpose equipment, which significantly increases the cost of producing the biosensors, thereby limiting their widespread availability in the marketplace.

The presence of multiple functions in a ferrocene‐based sensor also complicates manufacturing and can reduce the overall reliability of the biosensor [131] as well as complicate the quality control due to the increased sophistication of testing protocols required to ensure that each batch of production can perform consistently. As miniaturization continues to be a driving force behind the development of point‐of‐care biosensors, there continue to be challenges to maintaining sensor performance while reducing device dimensions and manufacturing tolerances [132].

### 3.2 Future Perspectives and Emerging Opportunities

Despite current limitations, several transformative developments indicate promising pathways for advancing ferrocene‐based sensor technologies. These emerging trends span technological innovations, application domains, and integration strategies that collectively participate in robust future for ferrocene‐based analytical platforms.

#### 3.2.1 Artificial Intelligence and Smart Sensor Systems

The use of artificial intelligence (AI)/machine learning is an entirely new way to develop both the evaluation of sensor output and sensor performance optimization. Ferrocene‐based sensors using AI‐assisted signal processing capabilities have the potential to address many of today's shortcomings, including real‐time calibration, predictive maintenance capabilities, and advanced sensor output analysis [133]. Machine learning algorithms may be used to detect and correct matrix effects, determine sensor degradation patterns, and develop measurement protocols for different samples or environments.

New generations of biosensors are being developed that include functionalized ferrocene derivatives that have better stability than previous versions of these biosensors and increased functionality [134]. Recently developed nanofibers containing ferrocene loaded into electrospun materials provide greater enzyme mediator surface area while preventing active ingredient leaching from the material. These advancements represent significant progress toward second‐generation biosensors with enhanced reliability and longer operational lifetimes [134].

#### 3.2.2 Wearable and Point‐of‐Care Technologies

The application of ferrocene sensors in the future will be strongly associated with wearable and portable devices to monitor the health status of individuals continuously and to provide personalized medicine. Wearable technology has demonstrated enormous potential to utilize noninvasive and continuous methods for multiple biomarkers at a single time, providing an overall vision of the status of health of an individual by means of a continuous analysis of physiological parameters [135, 136]. In this sense, advanced wearable platforms using ferrocene‐based sensing components are expected to have a high impact on the detection of glucose, lactates, electrolytes, and other clinically relevant analytes from biological samples, which can be easily obtained (e.g., from the sweat or from the interstitial fluid).

Hydrogel flexible sensors based on ferrocene represent an innovative approach for the detection of oxidative stress and for therapeutic monitoring [137]. In fact, these systems represent a combination of sensing and possible therapeutic properties, thus representing a new field of research for the creation of integrated diagnostic and treatment platforms for personalized health care.

#### 3.2.3 Digital Health Integration and Internet of Things (IoT) Connectivity

FBSs and the digital health environment can combine to deliver and manage care in a new way. Ferrocene‐based electrochemical sensors can be optimized using AI to ensure consistent and reliable measurements, thus allowing patients to monitor their health remotely through telemedicine applications and increasing access to healthcare services [138]. The combination of Internet of Things (IoT) and sensor technologies based on ferrocene allows for real‐time data transmission into the “cloud” for analysis and further supports integration into electronic health record systems. This will support individualized healthcare strategies as well as the ability to better manage population health [139].

#### 3.2.4 Novel Applications and Emerging Domains

A recent application of ferrocene derivatives is electrochemical sequencing, developing methods that use ferrocene‐containing oligourethane (ferrocene‐containing oligomers) for new analytical methods for polymer characterization and molecular identification [140]. Electrochemical sequencing uses differential pulse voltammetry and chain end degradation to produce temporal electrochemical fingerprints that represent the polymer sequence [140]. The ferrocene units served as both structural components and electroactive reporters, with each unit giving a unique signature in a voltammogram and correlating with sequence information [140].

As such, the ferrocene units present an unparalleled opportunity for advanced analytical applications, such as the quality control of polymers during synthesis, forensic identification of synthetic materials, and development of information‐storage systems using sequence‐defined polymers [140]. Due to its ability to be used in conjunction with micro‐scale electrochemical systems, it can provide great potential for developing portable analytical devices and field‐deployable applications.

### 3.3 Research Priorities and Strategic Development Needs

Future studies should focus on identifying ways to increase stability using innovative molecular designs to protect ferrocene derivatives from degradation mechanisms. The use of coating, encapsulation methods, and matrix materials may extend the life of sensors while maintaining analytical capability. Additionally, establishing standard processes for fabrication, performance measurement, and quality control is essential for FDA approval and clinical success.

Another key area of future study involves expanding the use of ferrocene‐based sensors to emerging biomarkers and environmental monitoring in line with broader trends in metallic nanoparticle‐enabled diagnostics and therapy. Development of novel ferrocene derivatives that are selective and sensitive for certain analytes could open a number of additional application areas, including potential unmet clinical diagnostics, environmental monitoring, or food safety analysis applications that would utilize sensors that measure glucose levels and/or detect heavy metals.

## 4 Challenges and Future Perspectives

While ferrocene can be utilized for several sensing applications, several obstacles exist that may hinder the commercialization of ferrocene‐based sensors on a larger scale. One of the main hurdles is the instability of the materials containing ferrocene when exposed to harsh conditions (high water content, acids, and biofluids), which have been proven to cause degradation of the ferrocene‐functionalized MOF structure, resulting in decreased long‐term sensing capability [33, 34, 35, 36, 37]. Another problem is the difficulty in creating large quantities of the same ferrocene‐based sensor material with consistent reproducibility and scalability. Examples of ferrocene‐based sensors typically consist of MOFs, nanoparticles, and/or multiple‐step surface functionalization [37, 38, 39, 40], resulting in a limitation in producing identical samples in large quantities. Therefore, the ability to create large quantities of the same material with high reproducibility will greatly contribute to the utilization of ferrocene‐based sensors in practical applications.

The development of highly selective, sensitive, stable, and reproducible sensors using ferrocene‐based redox chemistry is one of the most promising areas of electrochemistry. However, there are still several problems that must be resolved before this technology becomes a practical tool. The first problem is the performance of sensors in real‐world matrices (e.g., biological fluids, environmental pollutants, and agricultural products). Ferrocene‐based sensing systems have shown much potential in a lab setting but can be greatly affected by the many different types of matrix interference found in many real‐world matrices [44, 45, 46, 47], which leads to reduced selectivity and unreliability in signal generation. Secondly, due to the fact that carbon‐based ferrocene surfaces are generally less than perfectly functionalized [46, 47, 48] and vary from batch to batch, it has been challenging to create uniform and reproducible methods for quantitatively analyzing ferrocene‐based sensing systems. Therefore, addressing these challenges will be critical to developing ferrocene‐based sensors that will produce reliable and consistent results beyond the scope of small‐scale “proof of concept” type studies.

To continue to develop sensors that can produce reliable and consistent results, researchers will need to develop sensing systems that contain both high‐sensitivity and high‐stability ferrocene‐based sensing elements. In addition, researchers will need to develop new synthesis techniques for ferrocene‐based sensing systems that are cost‐effective and scalable. These new synthesis techniques should also be environmentally friendly so that they do not damage the environment when they are used [41, 42, 43, 45, 141].

Polymer‐based and carbon‐based sensing systems seem to be good candidates for meeting these requirements because of their mechanical flexibility, processability, and compatibility with wearable and portable sensing systems. Finally, the integration of emerging technologies (i.e., multianalyte detection, data‐assisted analysis, and smart sensing platforms) with ferrocene‐based sensors will likely expand their application and functionality.

In conclusion, overcoming the challenges identified earlier while maintaining the use of the unique redox chemistry of ferrocene will allow ferrocene‐based sensing systems to transition from being an area of research conducted primarily in the laboratory to a useful analytical technology in the real world. Optimizing materials, integrating devices, and creating applications will determine the next generation of ferrocene‐based sensors.

## 5 Conclusion

FBSs have many different uses today that include biosensing, biomedical diagnostics, environmental monitoring, and industrial analysis. Ferrocene has reversible redox (reduction‐oxidation) reactions, is chemically stable, and has tunable electronic properties; therefore, it can be used as an electrochemical mediator for highly accurate biosensing.

A wide variety of analytes can be detected using FBSs, including biomarkers, pollutants, and process control parameters. In recent years, many developments have occurred that will allow these sensors to continue to grow in complexity and innovation, including multiferrocene systems, metal‐organic frameworks, dual‐mode sensing, and electrochemical sequencing.

While there has been much development of FBSs, the use of these sensors on a large scale is still limited by the instability of ferrocene under physiological conditions, matrix effects in complex biological samples, and limitations to the commercialization of these sensors. Therefore, improving the long‐term stability of ferrocene and optimizing the design of ferrocene molecules are two important areas of study that need to be developed.

However, the future of ferrocene‐based sensors appears to be bright. Advances in artificial intelligence/machine learning, wearable electronics, and digital health will provide new methods for data processing/calibration and continuous monitoring of biosensors. The use of ferrocene‐based sensors will extend into many other emerging areas, such as medical imaging and other diagnostics, which further support the vast potential of ferrocene‐based sensor technologies. Therefore, the use of ferrocene‐based sensors in the development of advanced diagnostic and analytical instrumentation will be driven by advancements in the fields of material science, sensor design, and digital instrumentation.

## Conflicts of Interest

The authors declare no conflicts of interest.
